# The effect of anti-inflammatory drugs on ASC gene level and cellular viability

**DOI:** 10.1186/1546-0096-13-S1-P117

**Published:** 2015-09-28

**Authors:** B Balci Peynircioglu, YZ Akkaya Ulum, EZ Taskiran, M Arici, E Yilmaz

**Affiliations:** 1 Hacettepe University, Medical Biology, Ankara, Turkey; 2 Hacettepe University, Medical Genetics, Ankara, Turkey; 3 Hacettepe University, Nephrology, Ankara, Turkey

## Introduction

FMF is the most common of the autoinflammatory diseases and is characterized by recurrent attacks of fever and painful inflammation. Treatment of colchicine reduces the frequency and severity of FMF attacks. The FMF gene, MEFV, encodes a protein called pyrin, appears to be a regulator of inflammation through its interactions with several proteins that are related to regulation of cytokine secretion, cytoskeletal signaling and cell death. ASC (Apoptosis-associated Speck-like protein containing a Caspase recruitment domain) is a pyrin-interacting protein, which is a key adaptor component of the inflammasome. In this study, we hypothesized that many anti-inflammatory drugs with different mechanisms of action may have an effect on ASC gene expression level and cellular viability.

## Objectives

To determine the effect of colchicine, naproxen, prednol-l, acetylsalicylic acid, and azathioprine on ASC gene expression level and cellular viability using a monocytic cell line.

## Materials and methods

We used a differentiated monocytic cell line, THP-1 cells, which naturally express Pyrin interacting proteins. Cells were differentiated with PMA and treated with 100 ng/ml, 5 uM, 50 nmol/L, 600 uM, and 10 uM of colchicine, naproxen, prednol-l, acetylsalicylic acid, and azathioprine containing medium respectively, for 24 h. After qPCR was performed to measure ASC gene expression level in differentially treated cells. Student's t test was used for comparison of the means among groups. We used impedance-based xCELLigence Real-Time Cell Analysis detection platform, for real-time monitoring of cell viability of treated THP-1 cells. THP-1 cells were monitored for 24 h after treatment and electrical impedance, which is recorded as Cell Index (CI) values, reflected the biological statue of monitored cells' viability.

## Results

According to qPCR analysis; ASC gene expression was down regulated in colchicine (p<0.01), naproxen, prednol-L (p<0.01) and acetylsalicylic acid (p<0.001) treated cells. There was no change in gene expression in azathioprine treated cells. According to cell viability assay; CI values indicating the cells’ viability were increased in naproxen (p<0.05), prednol-L and acetylsalicylic acid (P<0.05) treated cells. We observed non-specific increase in CI values of colchicine and azathioprine treated cells.

## Conclusion

These anti-inflammatory drugs are known to have different mechanisms of action however they are all used to treat pain or inflammation. In order to understand whether these drugs’ therapeutic mechanism is related with ASC or not, we tested ASC gene expression level and cellular viability. Since ASC is a very well known proapoptotic protein, our results showing a decrease in gene expression and increase in cell viability suggested that naproxen, prednol-L and acetylsalicylic acid may have a therapeutic effect through ASC-inflammasome platform.

